# A Cross-Sectional Study: Association Between Nutritional Quality and Cancer Cachexia, Anthropometric Measurements, and Psychological Symptoms

**DOI:** 10.3390/nu17152551

**Published:** 2025-08-04

**Authors:** Cahit Erkul, Taygun Dayi, Melin Aydan Ahmed, Pinar Saip, Adile Oniz

**Affiliations:** 1 Department of Nutrition and Dietetics, Faculty of Health Sciences, Near East University, Mersin 99138, Turkey; dyt.cahit@gmail.com; 2 Department of Nutrition and Dietetics, Near East University Hospital, Mersin 99138, Turkey; 3 Department of Medical Oncology, Institute of Oncology, Istanbul University, Istanbul 34093, Turkey; melin.ahmed@istanbul.edu.tr (M.A.A.); pinarsaip@gmail.com (P.S.); 4 Department of Neurosciences, Institute of Graduate Studies, Near East University, Mersin 99138, Turkey; adile.oniz@neu.edu.tr; 5 Brain and Conscious States Research and Application Center, Near East University, Mersin 99138, Turkey

**Keywords:** cancer, cachexia, malnutrition, SGA, SCL-90-R

## Abstract

**Background/Objectives**: Cancer is a complex disease that affects patients’ nutritional and psychological status. This study aimed to assess the nutritional status of patients diagnosed with lung and gastrointestinal system cancers and evaluate its association with anthropometric measurements, nutrient intake, and psychological symptoms. **Methods**: This cross-sectional study was conducted with 180 patients with lung and gastrointestinal system cancers. Data were collected face-to-face by a questionnaire that included the Subjective Global Assessment-(SGA), Cachexia Assessment Criteria, 24 h Food Consumption Record, and Symptom Checklist-90-Revised-(SCL-90-R). Some anthropometric measurements were collected. **Results**: Body Mass Index (BMI) was found to be significantly lower (*p* < 0.001) in SGA-B (moderately malnourished) and SGA-C (severely malnourished) compared to those in SGA-A (well-nourished). The calf circumference was significantly lower (*p* = 0.002) in SGA-C compared to those in SGA-A and SGA-B. The mean SGA scores were found to be higher in cachexia-diagnosed participants (*p* < 0.001). The energy intake of SGA-C was significantly lower than SGA-A and SGA-B (*p* < 0.001). In addition, the energy intake of SGA-B was lower than SGA-A (*p* < 0.001). The protein intake of SGA-C was lower than SGA-A and SGA-B (*p* < 0.001). The protein intake of SGA-B was lower than SGA-A (*p* < 0.001). Regarding the intake of vitamins A, C, E, B1, and B6 and carotene, folate, potassium, magnesium, phosphorus, iron, and zinc, SGA-B and SGA-C were significantly lower than SGA-A (*p* < 0.001). Additionally, only phobic anxiety was found to be significantly higher in SGA-B than in SGA-A (*p*: 0.024). **Conclusions**: As the level of malnutrition increased, a reduction in some nutrient intake and anthropometric measurements was observed. No significant difference was found in any psychological symptoms except phobic anxiety. With this in mind, it is important that every cancer patient, regardless of the stage of the disease, is referred to a dietitian from the time of diagnosis.

## 1. Introduction

Cancer is the second leading cause of death worldwide. In 2022, approximately 20 million new cancer cases and nearly 10 million cancer-related deaths were reported [1]. Among the most common complications in cancer patients are nutritional disorders such as malnutrition and cachexia. Malnutrition affects 20–85% of cancer patients, depending on the diagnosis [2]. Disease-related malnutrition is defined as a condition resulting from the activation of systemic inflammation due to an underlying disease such as cancer. The inflammatory response can lead to anorexia, involuntary weight loss, changes in body composition, and a decline in physical function [3]. It is estimated that 10–20% of cancer-related deaths are more closely associated with malnutrition than malignancy itself. Therefore, nutrition is a crucial component of cancer treatment [4].

According to Fearon et al., cancer cachexia is characterized by the loss of muscle mass, with or without associated fat loss, which cannot be fully reversed by conventional nutritional therapy [5]. The prevalence of cachexia is higher in patients with gastrointestinal system (GIS) and lung cancer [6]. Studies have reported that the prevalence of cachexia in GIS and lung cancers ranges from 34.5% to 69% [7,8]. Malnutrition is particularly common among hospitalized patients and those with advanced or metastatic disease [9]. It has been reported that up to 80% of patients with GIS cancer may experience malnutrition [10]. Various factors contribute to malnutrition in gastrointestinal malignancies, including tumor-related factors such as obstruction or malabsorption, immune-related factors leading to anorexia or metabolic dysfunction, and treatment-related factors such as nausea, vomiting, and ostomy-related losses due to chemotherapy [11].

On the other hand, psychiatric disorders such as depression and anxiety are also common in cancer patients; however, they are often overlooked. The physical burden and significance of cancer, its treatment, and various biological, psychological, or social factors all contribute to the emergence of psychological symptoms [12]. Therefore, a comprehensive approach is needed—one that considers not only the physical condition but also the psychological well-being of patients with cancer cachexia. From this perspective, this study aimed to evaluate the effects of nutrition quality on cancer cachexia and psychological symptoms of adult patients aged 19–64 years (diagnosed with lung, esophageal, gastric, colon, and pancreatic cancer).

## 2. Materials and Methods

### 2.1. Study Setting, Timeframe, and Sample Selection

The study sample consisted of adult patients (aged 19–64 years) diagnosed with lung, esophageal, gastric, colon, or pancreatic cancer who were receiving treatment in the clinic and outpatient departments of the Istanbul University Oncology Institute. To standardize other natural physiological variables in the pediatric and geriatric age groups, this study focused on individuals within the adult age classification. For the ANOVA test conducted to compare four different groups (stages I, II, III, and IV), an effect size of 0.25, an alpha error rate of 5% (α: 0.05), and a power value of 80% (0.8) were used, determining that a sample size of 180 patients was sufficient for this study. With this in mind, 180 patients participated in the presented study, which is sufficient to ensure the representativeness of the results. Patients were selected using a random sampling method, and participation in this study was voluntary. Those who had psychiatric health problems and similarly those who were unable to complete the questionnaire were not included in this study.

### 2.2. Study Design and Data Collection Tools

This study was designed as a descriptive and cross-sectional study. A face-to-face data collection technique was employed, and a questionnaire was used as the data collection tool. The questionnaire consisted of five sections: general and clinical information, 24 h food consumption record, Subjective Global Assessment (SGA), Fearon et al.’s cachexia assessment criteria, and the Symptom Checklist-90-Revised (SCL-90-R) for psychological symptom screening. In addition, as the clinical background, certain blood parameters, diagnosis, and treatment methods of the patients were recorded, and their anthropometric measurements were determined.

### 2.3. General and Clinical Information

In the general information section, sociodemographic data such as age and gender, disease-related information (cancer type), and treatment type were assessed. Data on cancer type, stage, duration, applied treatment, and biochemical findings were obtained from patient records under the supervision of an oncologist. Blood parameter analyses, including C-reactive protein (CRP), neutrophils, albumin, lymphocytes, leukocytes, hemoglobin, and hematocrit, were conducted in the biochemistry laboratory of the university hospital where the study was carried out. The hospital’s reference values were used as the standard for evaluating biochemical parameters.

### 2.4. 24-h Food Consumption Record

To assess the patients’ food intake, all foods and beverages consumed in the past 24 h were retrospectively recorded. The data collection was conducted entirely by a single researcher, and to facilitate accurate portion estimation, the Food Photograph Catalog developed by Hacettepe University was used. The energy and nutrient content of each recorded food and beverage was calculated using the Computer-Assisted Nutrition Program, Nutrition Information Systems Package Program (BeBiS), a database specifically developed for Turkey.

### 2.5. Subjective Global Assessment (SGA)

In this study, the Subjective Global Assessment (SGA) test was used for nutritional screening. SGA has been validated and found to be reliable in identifying malnutrition as part of a comprehensive nutritional assessment in adult oncology patients [13,14]. The SGA includes additional questions related to the presence of common nutritional symptoms and body weight loss during cancer treatment. As the score increases, the risk of malnutrition also increases. Additionally, based on the SGA score, patients’ nutritional status is classified into three categories: well-nourished (A), moderately malnourished or suspected malnutrition (B), and severely malnourished (C). For each component of the SGA, scores ranging from 0 to 4 are given depending on the effect of the symptom on nutritional status. The higher the score, the higher the risk of malnutrition. A score higher than nine is considered to require nutritional intervention (severely malnourished), a score between two and eight is considered to require intervention by a dietitian together with a nurse or doctor based on symptom research (moderately malnourished), and a score between zero and one is considered to require no nutritional intervention (well-nourished) [15].

### 2.6. Cachexia Assessment Criteria

Patients were classified for cachexia based on the cachexia criteria developed by Fearon et al. According to this classification, pre-cachexia is defined as ≤5% body weight loss, anorexia, and metabolic alterations. Cachexia is defined as >5% body weight loss, or a BMI of <20 kg/m^2^ with >2% body weight loss, typically accompanied by reduced food intake or systemic inflammation. The CRP parameter was used when evaluating systemic inflammation. Terminal cachexia is characterized by a variable degree of cachexia, a pro-catabolic state, cancer that is unresponsive to treatment, low-performance status, and an expected survival of fewer than three months [5]. In our study, terminal cachexia was diagnosed by the oncologist.

### 2.7. Anthropometric Measurements

The anthropometric measurements of the patients were conducted by the researcher in accordance with standard protocols. Body height was measured using a wall-mounted stadiometer while the patients were barefoot, and the measurement was recorded in centimeters. During body height measurement, attention was given to ensuring that the feet were together and that the patients were positioned in the Frankfort plane. Body weight, body fat mass, fat-free mass, body fat percentage, and total body water mass were measured using the Tanita BC 418 bioelectrical impedance analysis (BIA) device. The device was calibrated, and all measurements were performed according to the manufacturer’s instructions.

The BIA device analyzed the body in five separate segments and provided measurements with an accuracy of 0.1 kg. Measurements were taken while patients were fasting after they had emptied their bladder, and with no metal objects on their bodies. Additionally, for female patients of reproductive age, measurements were taken outside the premenstrual, menstrual, and postmenstrual periods to ensure accuracy.

### 2.8. Symptom Checklist-90-Revised (SCL-90-R)

The Symptom Checklist-90-Revised (SCL-90-R) is a self-report psychiatric screening tool that allows individuals to assess themselves, and its final version was developed by Derogatis (1977). The scale consists of 90 items categorized into ten subdimensions: Obsessive-Compulsive Disorder (OCD), Somatization, Interpersonal Sensitivity, Depression, Anxiety, Hostility, Phobic Anxiety, Paranoia, Psychoticism, and Additional Items. The additional items section includes symptoms such as eating and sleep disorders and feelings of guilt [16]. The validity and reliability of the scale for Turkey were established by Dağ (1991), and its Cronbach’s alpha (internal consistency coefficient) was found to be 0.97. The scale is based on a five-point Likert scale (0: Not at all, 1: A little, 2: Moderate, 3: Quite a bit, 4: Extremely). Scores are interpreted as follows: 0.00–1.50 is considered normal, 1.51–2.50 indicates a high level of distress, and 2.51–4.00 indicates a very high level of distress [17].

### 2.9. Statistical Analysis

Descriptive statistics were initially performed for the study variables. For quantitative variables, the mean ($\bar{x}$), standard deviation (SD), minimum value, maximum value, and median were calculated. For qualitative data, frequency (n) and percentage (%) values were determined. The distribution characteristics of quantitative variables across groups were examined using the Kolmogorov–Smirnov or Shapiro–Wilk normality tests, and the assumptions for parametric tests were assessed. Since the assumptions for parametric tests were not met, non-parametric statistical methods were applied. For comparisons between two independent groups, the Mann–Whitney U test was used. When the number of independent groups exceeded two, the Kruskal–Wallis test was performed first, and if statistical significance was observed, pairwise comparisons were conducted using the Mann–Whitney U test. The statistical significance level for the entire study was set at 0.05. All statistical analyses were performed using the Statistical Package for the Social Sciences (SPSS-Version 26.0 for Mac).

## 3. Results

Among the participants, 33.30% were women and 66.70% were men, with an overall mean age of 57.42 ± 8.03 years. Regarding cancer types, 36.70% of the patients had lung cancer, 4.40% had esophageal cancer, 25.60% had gastric cancer, 10.60% had pancreatic cancer, and 22.80% had colon cancer.

When evaluating cancer stages, 22.20% of the patients were at stage 1, 43.30% at stage 2, 16.70% at stage 3, and 17.80% at stage 4. Regarding treatment types, 90.00% of the patients were receiving chemotherapy, 3.90% were undergoing both chemotherapy and radiotherapy, 5.00% were receiving surgery and chemotherapy, and 1.10% were undergoing immunotherapy. In terms of the distribution of medications used, 59.40% were antimetabolites, 8.40% were biological agents, 14.40% were platinum-based compounds, and 1.10% were immunotherapy drugs. The mean, standard deviation, median, minimum, and maximum values for the number of treatment cycles received by the patients are presented in Table 1.

Among the patients, 15.00% were classified as pre-cachexia, 15.00% as cachexia, and 4.40% as terminal cachexia. When evaluating the Subjective Global Assessment (SGA) scores, 18.90% of the participants were categorized as SGA-B and 11.70% as SGA-C (Table 2).

Table 3 shows the mean, standard deviation, median, and min–max values of the individuals participating in the study according to SCL-90R scores. Accordingly, the overall score was found to be 1.54 ± 0.31 (0.74–2.31). The subscale scores are presented in Table 3.

**Figure 1:**
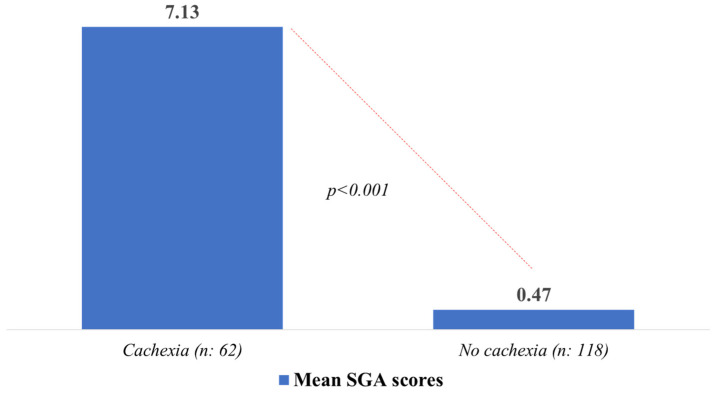
Patient’s Mean SGA Scores According to Cachexia Status [analyzed using the Mann–Whitney U test].

As shown in Figure 1, participants diagnosed with cachexia had higher mean SGA scores, indicating poorer nutritional status.

Effects of nutritional quality on the anthropometric measurements are shown in Table 4. Accordingly, the BMI value was significantly lower (*p* < 0.001) in patients classified into the SGA-B and C categories compared to those in the SGA-A category. The calf circumference was significantly lower (*p*: 0.002) in patients classified in the SGA-C category compared to those in the SGA A and B categories. Regarding muscle mass and body water variables, the measurements of patients in the SGA-B category were significantly lower than those of patients in the SGA-A category (*p*: 0.007 for each).

When examining biochemical findings, the CRP variable was found to be significantly higher in patients classified in the SGA-C category compared to those in the SGA-A and SGA-B categories (*p* < 0.001). For the albumin variable, measurements of patients in the SGA-C category were significantly lower than those in the SGA-A and B categories (*p*: 0.002). No difference was found between patients in categories SGA-A and B. For the neutrophil variable, measurements of patients in the SGA-C category were significantly higher than those in the SGA-B category (*p*: 0.014). Regarding the leukocyte variable, measurements of patients in the SGA-B category were significantly lower than those in the SGA-A category (*p*: 0.005, Table 5).

Evaluation of patients’ energy and nutrient intake revealed that energy intake by patients in the SGA-C category was significantly lower than those in the SGA-A and SGA-B categories (*p* < 0.001). In addition, the energy intake by patients in the SGA-B category was also lower than those in the SGA-A category (*p* < 0.001). The water intake by patients in the SGA-B and SGA-C categories was significantly lower than those in the SGA-A category (*p* < 0.001). The protein intake by patients in the SGA-C category was lower than those in the SGA-A and SGA-B categories (*p* < 0.001). Similarly, the protein intake by patients in the SGA-B category was also lower than those in the SGA-A category (*p* < 0.001). The fat intake by those in the SGA-C category was significantly lower than those in the SGA-A and SGA-B categories (*p* < 0.001). Again, the fat intake by those in the SGA-B category was also lower than those in the SGA-A category (*p* < 0.001). In terms of the percentage (%) of fat intake, for patients in the SGA-C category it was significantly lower than for patients in the SGA-A category (*p*: 0.032). Carbohydrate intake by patients in the SGA-C category was significantly lower than by those in the SGA-A category (*p* < 0.001). However, in terms of the percentage (%) of carbohydrate intake, for patients in the SGA-C category it was found to be significantly higher than for those in the SGA-A category (*p*: 0.012). Polyunsaturated fatty acids (PUFA) intake by patients in the SGA-C category was significantly lower than for those in the SGA-A category (*p* < 0.001). Cholesterol intake by patients in the SGA-C category was significantly lower than for those in the SGA-A and SGA-B categories (*p* < 0.001). Regarding the intake of vitamins A, C, E, B1, and B6, and carotene, folate, potassium, magnesium, phosphorus, iron, and zinc, measurements of patients in the SGA-B and SGA-C categories were significantly lower compared to those in the SGA-A category (*p* < 0.001). Sodium intake by patients in the SGA-C category was significantly lower than those in the SGA-A category (*p* < 0.001). Calcium intake by patients in the SGA-C category was lower than those in the SGA-A and SGA-B categories. In addition, the calcium intake by patients in the SGA-B category was significantly lower than those in the SGA-A category (*p* < 0.001) (Table 6).

When the SCL-90R scale and subscale scores are examined, phobic anxiety was found to be higher in patients in the SGA-B category than for those in the SGA-A category (*p*: 0.024). No significant difference was found in any other behavioral or psychological variables (Table 7).

## 4. Discussion

In this study, both physical and psychological parameters were assessed using data from 180 patients undergoing cancer treatment and follow-up in the oncology unit, collected through face-to-face interviews and anthropometric measurements. The results highlight a critical interplay between cachexia, nutritional intake, and psychological factors.

Cancer is characterized by systemic inflammation, which, along with treatment-related side effects, leads to reduced food intake, anthropometric changes, and consequently, an increased risk of malnutrition, particularly cachexia, as well as negative effects on mood. It is well known that these issues become more prevalent as the cancer stage progresses [18,19]. Based on the existing literature, this study aimed to identify differences in anthropometric measurements, nutrient intake, laboratory findings, psychological symptoms, and other parameters that influence nutritional status in patients diagnosed with lung and GIS cancers.

Malnutrition is one of the most common symptoms of cancer and one of the serious side effects of treatment. Early diagnosis of malnutrition is vital to reduce the risk of complications, improve clinical outcomes, and reduce healthcare costs. BMI and calf circumference are useful clinical indicators of nutritional status because they are easy to determine and inexpensive. Lower BMI and calf circumference have been suggested as good measures to identify malnutrition [20].

In a study conducted by Yin et al. on patients with esophageal cancer, BMI was found to be 20.59 ± 2.78 kg/m^2^ in malnourished patients, whereas it was 22.90 ± 7.58 kg/m^2^ in non-malnourished patients. In the same study, calf circumference was 31.94 ± 3.26 cm in malnourished patients and 33.93 ± 16.01 cm in non-malnourished patients [21]. In another study by Yin et al., BMI was 21.40 ± 3.14 kg/m^2^ in malnourished patients and 23.74 ± 3.33 kg/m^2^ in non-malnourished patients. Similarly, calf circumference was 32.23 ± 3.49 cm in malnourished patients and 34.29 ± 3.77 cm in non-malnourished patients [22]. Consistent with these studies, in our study, the BMI value was found to be significantly lower (*p* < 0.001) in patients classified into the SGA-B (23.82 ± 3.85) and C (21.56 ± 3.56) categories compared to those in the SGA-A (25.89 ± 3.14) category. The calf circumference was found to be significantly lower (*p*: 0.002) in patients classified into the SGA-C (33.43 ± 2.99) category compared to those in the SGA-A (36.06 ± 3.09) and B (35.88 ± 3.57) categories (Table 4).

BIA is a widely used method in studies evaluating body composition (fat mass, lean mass, and total body water). It estimates the distribution of body fluids in intracellular and extracellular spaces, in addition to body components. This technique involves the passage of a painless, low-amplitude electrical current through wires connected to electrodes or conductive surfaces in direct contact with the skin [23]. In a study conducted by Mueller et al. on GIS cancer, muscle and fat mass values measured using BIA were found to be significantly lower in malnourished patients (9.68, 5.09) compared to non-malnourished patients (10.64, 6.59) [24]. Similarly, in our study, muscle (%) and water (%) measurements in patients classified into the SGA-B category (51.87 ± 9.42, 39.70 ± 7.95) were found to be significantly lower than those in the SGA-A category (57.01 ± 9.10, 43.93 ± 7.17) (*p*: 0.007; Table 4).

In another study conducted by Yin et al., CRP levels were found to be higher in malnourished patients (27.88 ± 35.99 mg/L) compared to non-malnourished patients (13.94 ± 17.94 mg/L). Similarly, white blood cell count (mm^3^) was higher in malnourished patients (7.11 ± 3.93) than in non-malnourished patients (6.63 ± 7.22). On the other hand, albumin levels (g/L) were lower in malnourished patients (36.96 ± 5.75) compared to non-malnourished patients (40.32 ± 7.68) [25]. In a study conducted by Li et al. on lung cancer patients, lymphocyte (1.35 ± 0.07) and albumin (40.08 ± 0.74) levels were found to be significantly lower in patients in the SGA-C category compared to those in the SGA-A and SGA-B categories [26]. Similarly, in our study, CRP levels were significantly higher in patients classified into the SGA-C category compared to those in the SGA-A and SGA-B categories (*p* < 0.001). For the albumin variable, it was significantly higher in patients classified into the SGA-B and A categories than in those in the SGA-C category (*p*: 0.002; Table 5).

Cancer-related malnutrition results from insufficient nutrient intake due to the systemic effects of the disease, the adverse impacts of cancer and its treatment. Several factors can increase the risk or severity of malnutrition, including tumor location (head and neck or gastrointestinal), symptoms (e.g., loss of appetite, early satiety, and fatigue), treatment methods (surgery, chemotherapy, or radiotherapy), complications (e.g., mucositis, nausea, taste alterations), and psychological distress [27]. The goal of nutritional therapy is to maintain oral nutrition by minimizing nutrition-related discomfort and maximizing the enjoyment of food through strategies such as diet counseling, nutritional supplementation, and oral nutritional supplements (ONS) provided by a dietitian [28]. Cancer patients often fail to meet the recommended protein intake. According to the ESPEN guidelines, a higher protein intake range (1.2–1.5 g/kg/day) is required, and 2.0 g/kg/day is associated with a positive protein balance. Additionally, it is recommended that carbohydrates account for 55–65% of non-protein energy intake, while fat intake should be 35–45% [29]. Due to the negative effects of active treatment and inadequate nutrition in many patients, the American Institute for Cancer Research (AICR), the American Cancer Society (ACS), and ESPEN support the use of multivitamin-multimineral supplements at doses close to the daily dietary intake recommendations. However, in the absence of specific deficiencies, high-dose vitamin and mineral supplementation is not recommended [30]. In a study by Kaya and Pekcan on patients with GIS cancer, no statistically significant differences were found in energy and nutrient intake among the patients [31]. There is no similar study on this subject in the literature. In our study, it was observed that the intake of energy, macro- and micronutrients was significantly lower in patients with poorer nutritional status (SGA-C) compared to those in better nutritional categories (SGA-A and SGA-B). Particularly in the SGA-C group, the intake of energy, water, protein, fat, cholesterol, certain fatty acids, sodium, and carbohydrates was lower. Moreover, the intake of various micronutrients, including vitamins (A, B_1_, B_6_, C, E, folate, and carotene) and minerals (calcium, potassium, magnesium, phosphorus, iron, and zinc), was markedly reduced in the SGA-B and SGA-C groups compared to the SGA-A group. On the other hand, the percentage of carbohydrate intake within total energy was higher in the SGA-C group (Table 6).

In a study conducted by İlce et al. on cancer patients, the SCL-90R scores were reported as follows: somatization (1.04 ± 0.83), obsessive-compulsive disorder (0.81 ± 0.74), interpersonal sensitivity (0.70 ± 0.78), depression (0.86 ± 0.76), anxiety (0.70 ± 0.79), hostility (0.72 ± 0.76), phobic anxiety (0.36 ± 0.53), paranoid thoughts (0.56 ± 0.63), additional subscale score (1.07 ± 0.79), and overall scale score (0.75 ± 0.64) [32]. In another study conducted by Sharma and Sharma on lung cancer patients, the SCL-90R scores were reported as follows: somatization (1.58 ± 0.67), obsessive-compulsive disorder (1.62 ± 0.60), interpersonal sensitivity (1.44 ± 0.55), depression (1.70 ± 0.68), anxiety (1.71 ± 0.67), hostility (0.08 ± 0.28), phobic anxiety (1.07 ± 0.62), paranoid thoughts (1.45 ± 0.59), and psychoticism (1.44 ± 0.67) [33]. In our study, the somatization score was 2.74 ± 0.76, OCD score 1.29 ± 0.42, interpersonal sensitivity score 0.93 ± 0.40, depression score 2.84 ± 0.85, anxiety score 1.06 ± 0.38, hostility score 0.87 ± 0.43, phobic anxiety score 0.68 ± 0.44, paranoid thoughts score 0.79 ± 0.45, and psychoticism score 0.99 ± 0.37. The additional subscale and overall scores were found to be 1.86 ± 0.36 and 1.54 ± 0.31, respectively (Table 3).

## 5. Conclusions

In conclusion, this study clearly shows that malnutrition in cancer patients has serious negative effects not only on physical but also on anthropometric measurements, biochemical indicators and nutrient intakes. SGA-C patients have lower BMI, calf circumference, muscle mass, body water, energy and most micro/macronutrient intakes than SGA-A and SGA-B patients. One of the strengths of our study is that, unlike previous studies in the literature on lung and GIS cancer patients with cachexia and malnutrition, no study has been conducted with such detailed dietary intake records, nutrient assessments, and the application of the SCL-90R test as in our research. However, the limitations of our study include the absence of the Patient-Generated Subjective Global Assessment (PG-SGA) test, the use of albumin instead of pre-albumin due to its high cost, and that it was conducted only on outpatient or mobile patients; patients requiring clinical follow-up were not included. Although reminder visual materials were used by the researcher, the 24 h dietary recall method is based on memory and self-reporting, and therefore may be considered a limitation of this study.

## Figures and Tables

**Table 1:** Distribution of Patients’ Age, Gender, Cancer-Related Characteristics, and Number of Treatment Cycles Received.

		$\bar{x}$ ± SD	Median (Min–Max)
Age		57.42 ± 8.03	61.00 (25.00–64.00)
		Frequency (n)	Percentage (%)
Gender	Female	60	33.30
	Male	120	66.70
Cancer Type	Lung	66	36.70
	Esophageal	8	4.40
	Gastric	46	25.60
	Pancreas	19	10.60
	Colon	41	22.80
Cancer Stage	Stage 1	40	22.20
	Stage 2	78	43.30
	Stage 3	30	16.70
	Stage 4	32	17.80
Treatment Type	Chemotherapy	162	90.0
	Chemotherapy + Radiotherapy	7	3.90
	Surgery + Chemotherapy	9	5.0
	Immunotherapy	2	1.10
Medications Used	Antimetabolite	107	59.40
	Biological Agent	15	8.40
	Platinum-Based Compound	26	14.40
	Immunotherapy Drug	2	1.10
Number of Treatment Cycles Received		$\bar{x}$ ± SD	Median (Min–Max)
		2.63 ± 1.66	2.00 (1.00–9.00)

**Table 2:** Distribution of Cachexia Classification, SGA Scores, Descriptive Statistics of SGA Scores.

		Frequency (n)	Percentage (%)
Cachexia			
	No Cachexia	118	65.60
	Pre-cachexia	27	15.00
	Cachexia	27	15.00
	Terminal Cachexia	8	4.40
SGA Score			
A	Well nourished	125	69.40
B	Mildly/moderately malnourished	34	18.90
C	Severely malnourished	21	11.70

**Table 3:** Descriptive Statistics of Patients According to SCL-90R Scores.

	$\bar{x}$ ± SD	Median (Min–Max)
Somatization	2.74 ± 0.76	2.83 (1.00–4.00)
OCD	1.29 ± 0.42	0.30 (0.40–2.40)
Interpersonal Sensitivity	0.93 ± 0.40	0.89 (0.22–2.22)
Depression	2.84 ± 0.85	3.00 (1.00–4.00)
Anxiety	1.06 ± 0.38	1.00 (0.10–2.20)
Hostility	0.87 ± 0.43	0.83 (0.00–2.17)
Phobic Anxiety	0.68 ± 0.44	0.71 (0.00–2.43)
Paranoid Thoughts	0.79 ± 0.45	0.67 (0.00–2.33)
Psychoticism	0.99 ± 0.37	1.00 (0.20–2.10)
Additional Subscale	1.86 ± 0.36	1.86 (0.71–3.00)
Overall Score	1.54 ± 0.31	1.54 (0.74–2.31)

**Table 4:** Association Between Nutritional Status Classification and Mean Anthropometric Characteristics According to SGA Group.

SGA								
		A (n = 125)		B (n = 34)		C (n = 21)		*p* Value ^c^
		$\bar{x}$ ± SD	Median (Min–Max)	$\bar{x}$ ± SD	Median (Min–Max)	$\bar{x}$ ± SD	Median (Min–Max)	
Anthropometric Characteristics	BMI (kg/m^2^)	25.89 ± 3.14	25.20 (18.80–37.50)	23.82 ± 3.85	23.45 ^a^ (16.60–35.90)	21.56 ± 3.56	20.70 ^a^ (15.10–27.60)	<0.001
	Calf Circumference (cm)	36.06 ± 3.09	35.00 (26.00–43.00)	35.88 ± 3.57	35.00 (30.00–49.00)	33.43 ± 2.99	33.00 ^a,b^ (29.00–40.00)	0.002
	Fat (%)	24.63 ± 5.58	23.70 (11.30–44.00)	23.44 ± 6.50	22.60 (9.80–38.40)	20.87 ± 8.57	21.40 (10.40–44.70)	0.055
	Muscle Mass (%)	57.01 ± 9.10	56.30 (34.50–74.50)	51.87 ± 9.42	53.45 ^a^ (37.50–77.60)	53.29 ± 8.05	54.40 (35.00–67.40)	0.007
	Body Water (%)	43.93 ± 7.17	44.70 (25.30–55.70)	39.70 ± 7.95	40.90 ^a^ (27.30–56.80)	41.99 ± 8.00	43.40 (26.20–56.40)	0.007

a: Statistically significant difference relative to Group A (*p* < 0.05); b: Statistically significant difference relative to Group B (*p* < 0.05); c: Kruskal–Wallis test and Mann–Whitney U test.

**Table 5:** Association Between Nutritional Status Classification and Mean Blood Parameters According to SGA Group.

SGA								
		A (n = 125)		B (n = 34)		C (n = 21)		*p* Value ^c^
		$\bar{x}$ ± SD	Median (Min–Max)	$\bar{x}$ ± SD	Median (Min–Max)	$\bar{x}$ ± SD	Median (Min–Max)	
Biochemical Findings	CRP (mg/L)	11.72 ± 18.66	4.14 (0.28–88.10)	26.88 ± 47.90	5.54 (0.33–221.33)	110.71 ± 97.38	113.93 ^a,b^ (0.51–395.97)	<0.001
	Neutrophils (×10^9^/L)	5.29 ± 11.19	3.20 (0.53–96.20)	3.69 ± 1.55	3.58 (1.00–7.02)	6.58 ± 4.88	4.76 ^b^ (1.09–19.20)	0.014
	Albumin (g/dL)	4.04 ± 0.58	4.10 (1.70–5.10)	4.30 ± 1.24	4.17 (3.01–10.90)	3.61 ± 0.55	3.66 ^a,b^ (2.51–4.73)	0.002
	Lymphocytes (×10^9^/L)	2.49 ± 7.53	1.70 (0.10–85.10)	1.69 ± 0.86	1.50 (0.63–4.40)	1.98 ± 2.76	1.28 (0.30–13.71)	0.268
	Leukocytes (×10^3^/µL)	16.61 ± 7.35	16.27 (0.00–62.92)	13.47 ± 6.57	12.82 ^a^ (1.57–30.97)	12.48 ± 8.75	10.60 (1.41–28.48)	0.005
	Hemoglobin (g/dL)	11.73 ± 2.70	11.50 (7.90–35.10)	13.80 ± 13.52	11.60 (9.20–90.00)	11.38 ± 1.78	11.00 (8.20–15.10)	0.839
	Hematocrit (g/dL)	34.92 ± 4.89	34.90 (25.10–45.70)	34.82 ± 3.45	35.45 (28.70–42.60)	34.35 ± 4.69	33.80 (25.00–43.90)	0.854

a: Statistically significant difference relative to Group A (*p* < 0.05); b: Statistically significant difference relative to Group B (*p* < 0.05); c: Kruskal–Wallis test and Mann–Whitney U test.

**Table 6:** Association Between Nutritional Status Classification and Mean Energy and Nutrient Intake According to SGA Group.

SGA								
		A (n = 125)		B (n = 34)		C (n = 21)		*p* Value ^c^
		$\bar{x}$ ± SD	Median (Min–Max)	$\bar{x}$ ± SD	Median (Min–Max)	$\bar{x}$ ± SD	Median (Min–Max)	
Energy and Nutrient Intake	Energy (kcal)	1494.49 ± 412.30	1497.48 (392.87–3226.66)	1216.44 ± 490.53	1118.40 ^a^ (363.63–2331.08)	850.15 ± 411.69	682.22 ^a,b^ (337.42–1682.18)	<0.001
	Water (L)	789.72 ± 264.42	797.00 (136.46–1430.83)	659.50 ± 257.37	624.35 ^a^ (256.18–1186.56)	533.54 ± 282.89	505.46 ^a^ (187.43–1258.35)	<0.001
	Protein (g)	57.18 ± 17.94	57.69 (9.24–109.40)	47.16 ± 19.79	44.29 ^a^ (16.09–89.75)	30.90 ± 16.66	26.10 ^a,b^ (11.38–71.11)	<0.001
	Protein (%)	15.98 ± 4.03	15.00 (9.00–30.00)	16.47 ± 5.00	15.00 (11.00–33.00)	14.86 ± 2.39	14.00 (10.00–20.00)	0.625
	Fat (g)	79.42 ± 27.15	79.64 (19.52–179.16)	62.71 ± 29.84	57.73 ^a^ (10.16–125.28)	40.08 ± 22.26	34.17 ^a,b^ (10.32–83.23)	<0.001
	Fat (%)	47.24 ± 8.78	47.00 (25.00–71.00)	45.76 ± 10.94	48.00 (25.00–62.00)	41.14 ± 10.07	44.00 ^a^ (24.00–60.00)	0.032
	Carbohydrates (g)	134.42 ± 49.87	132.11 (9.27–312.22)	112.96 ± 55.72	109.61 (22.90–272.74)	89.07 ± 42.49	96.03 ^a^ (27.86–171.54)	<0.001
	Carbohydrates (%)	36.74 ± 9.78	36.00 (6.00–60.00)	37.76 ± 8.65	36.50 (19.00–56.00)	44.05 ± 10.90	43.00 ^a^ (22.00–65.00)	0.012
	PUFAs (g)	10.75 ± 7.43	8.86 (1.74–42.41)	8.58 ± 6.98	6.81 (1.44–27.61)	5.83 ± 3.41	4.28 ^a^ (1.50–15.39)	<0.001
	Cholesterol (mg)	376.66 ± 193.83	387.90 (32.00–1027.45)	307.64 ± 191.89	384.30 (67.20–803.35)	163.85 ± 180.53	96.65 ^a,b^ (14.70–597.15)	<0.001
	Vitamin A (mcg)	1203.16 ± 2142.69	870.20 (126.40–24,159.55)	673.85 ± 400.93	624.10 ^a^ (99.63–2035.59)	502.52 ± 476.47	585.36 ^a^ (23.85–1838.81)	<0.001
	Carotene (mg)	2.55 ± 2.77	1.38 (0.13–14.11)	1.23 ± 1.24	0.92 ^a^ (0.11–5.08)	1.38 ± 1.86	0.73 ^a^ (0.02–6.92)	<0.001
	Vitamin E (mg)	11.55 ± 6.81	9.74 (1.30–31.99)	8.32 ± 6.24	6.58 ^a^ (1.96–24.52)	6.55 ± 4.24	4.56 ^a^ (0.73–17.23)	<0.001
	Vitamin B_1_ (mg)	0.75 ± 0.28	0.71 (0.10–2.20)	0.59 ± 0.28	0.58 ^a^ (0.13–1.25)	0.49 ± 0.35	0.47 ^a^ (0.10–1.41)	<0.001
	Vitamin B_2_ (mg)	1.30 ± 0.57	1.26 (0.25–5.51)	0.96 ± 0.40	0.98 ^a^ (0.27–1.66)	0.67 ± 0.45	0.54 ^a^ (0.09–1.56)	<0.001
	Vitamin B_6_ (mg)	1.12 ± 0.43	1.06 (0.10–2.50)	0.83 ± 0.37	0.84 ^a^ (0.13–1.43)	0.65 ± 0.54	0.43 ^a^ (0.12–1.94)	<0.001
	Folate (mg)	248.29 ± 107.36	243.10 (47.35–882.63)	172.32 ± 72.91	178.47 ^a^ (42.70–307.10)	148.63 ± 117.43	125.92 ^a^ (19.60–392.50)	<0.001
	Vitamin C (mg)	93.20 ± 56.79	85.62 (0.00–258.35)	62.42 ± 49.32	53.51 ^a^ (0.21–184.59)	53.83 ± 67.41	21.19 ^a^ (0.25–258.35)	<0.001
	Sodium (mg)	3241.96 ± 6332.60	2546.95 (458.40–52,217.20)	2082.95 ± 750.59	2034.67 (676.95–3555.50)	1701.52 ± 1285.98	1296.12 ^a^ (496.65–6221.31)	<0.001
	Potassium (mg)	1951.07 ± 702.14	1917.59 (199.70–5659.15)	1407.40 ± 677.26	1449.18 ^a^ (242.00–3212.30)	1202.72 ± 861.00	1063.44 ^a^ (207.60–3022.30)	<0.001
	Calcium (mg)	634.91 ± 243.32	628.78 (74.25–1370.90)	497.78 ± 224.03	538.87 ^a^ (127.90–1058.40)	345.76 ± 219.16	313.15 ^a,b^ (34.40–764.51)	<0.001
	Magnesium (mg)	224.74 ± 88.70	226.00 (34.54–729.74)	172.62 ± 81.03	169.03 ^a^ (45.21–407.55)	145.96 ± 103.12	142.78 ^a^ (28.55–370.81)	<0.001
	Phosphorus (mg)	951.08 ± 337.06	916.40 (211.95–2198.55)	748.98 ± 307.48	731.62 ^a^ (241.40–1448.90)	550.81 ± 358.88	438.35 ^a^ (129.85–1486.05)	<0.001
	Iron (mg)	8.18 ± 3.25	7.68 (1.49–24.69)	5.82 ± 2.43	5.24 ^a^ (2.05–12.05)	4.95 ± 3.92	5.18 ^a^ (1.22–15.25)	<0.001
	Zinc (mg)	8.76 ± 3.20	8.04 (1.78–18.89)	6.70 ± 2.31	6.71 ^a^ (2.05–11.24)	4.81 ± 3.27	3.56 ^a^ (1.04–11.94)	<0.001
	Vitamin B_12_ (mg)	4.13 ± 6.76	3.06 (0.20–74.15)	3.56 ± 2.24	3.14 (0.35–9.88)	4.22 ± 2.69	3.63 (0.40–10.74)	0.625
	Selenium (mg)	10.55 ± 9.86	13.77 (0.00–49.92)	16.52 ± 19.81	16.29 (0.00–115.21)	14.45 ± 14.56	16.34 (0.00–48.42)	0.108

a: Statistically significant difference relative to Group A (*p* < 0.05); b: Statistically significant difference relative to Group B (*p* < 0.05); c: Kruskal–Wallis test and Mann–Whitney U test.

**Table 7:** Association Between Nutritional Status Classification and Mean SCL-90R Subscores According to SGA Group.

SGA								
		A (n = 125)		B (n = 34)		C (n = 21)		*p* Value ^c^
		$\bar{x}$ ± SD	Median (Min–Max)	$\bar{x}$ ± SD	Median (Min–Max)	$\bar{x}$ ± SD	Median (Min–Max)	
SCL-90 Score	Somatization	2.74 ± 0.78	2.83 (1.08–4.00)	2.71 ± 0.73	2.83 (1.00–4.00)	2.76 ± 0.69	2.83 (1.92–3.92)	0.842
	OCD	1.30 ± 0.43	1.30 (0.40–2.40)	1.27 ± 0.41	1.20 (0.40–2.20)	1.31 ± 0.44	1.30 (0.40–2.00)	0.859
	Interpersonal Sensitivity	0.89 ± 0.38	0.89 (0.22–2.22)	1.00 ± 0.41	1.00 (0.33–2.00)	1.07 ± 0.48	1.11 (0.44–2.11)	0.162
	Depression	2.83 ± 0.89	3.00 (1.00–4.00)	2.86 ± 0.78	3.00 (1.00–4.00)	2.86 ± 0.73	3.00 (1.92–4.00)	0.986
	Anxiety	1.05 ± 0.36	1.00 (0.10–2.10)	1.13 ± 0.45	1.10 (0.50–2.20)	1.04 ± 0.35	1.00 (0.30–1.70)	0.704
	Hostility	0.84 ± 0.42	0.83 (0.00–2.17)	0.91 ± 0.46	0.83 (0.33–2.00)	0.97 ± 0.42	1.17 (0.17–1.67)	0.365
	Phobic Anxiety	0.62 ± 0.40	0.57 (0.00–2.29)	0.87 ± 0.51	0.71 (0.00–2.43) ^a^	0.72 ± 0.43	0.71 (0.00–1.57)	0.024
	Paranoid Thoughts	0.76 ± 0.42	0.67 (0.00–1.83)	0.77 ± 0.45	0.67 (0.17–1.83)	0.99 ± 0.57	0.83 (0.17–2.33)	0.207
	Psychoticism	0.95 ± 0.36	0.90 (0.20–2.00)	1.12 ± 0.40	1.10 (0.40–2.10)	1.01 ± 0.38	1.00 (0.30–1.70)	0.072
	Additional Subscale	1.85 ± 0.37	1.86 (0.71–2.71)	1.87 ± 0.29	1.86 (1.29–2.43)	1.90 ± 0.39	1.86 (1.29–3.00)	0.910
	Overall Score	1.52 ± 0.32	1.53 (0.74–2.31)	1.58 ± 0.29	1.62 (0.91–2.04)	1.59 ± 0.31	1.59 (1.08–2.19)	0.439

a: Statistically significant difference relative to Group A (*p* < 0.05); c: Kruskal–Wallis test and Mann–Whitney U test.

## Data Availability

The dataset used in this study is available on request.

## Abbreviations

The following abbreviations are used in this manuscript:

ACS	American Cancer Society
AICR	American Institute for Cancer Research
BIA	Bioelectrical Impedance Analysis
BMI	Body Mass Index
CRP	C-Reactive Protein
ESPEN	European Society for Clinical Nutrition and Metabolism
GIS	Gastrointestinal System
BeBiS	Nutrition Information Systems Package Program
OCD	Obsessive-Compulsive Disorder
PG-SGA	Patient-Generated Subjective Global Assessment
PUFA	Polyunsaturated Fatty Acids
SCL90R	Symptom Checklist-90-Revised
SD	Standard Deviation
SGA	Subjective Global Assessment
SPSS	Statistical Package for the Social Sciences
